# Diagnostic Potential of Metabolomic and Proteomic Biomarkers in Cardiology—A Narrative Review

**DOI:** 10.3390/biomedicines14020257

**Published:** 2026-01-23

**Authors:** Lazzat Zhussupbekova, Dinara Nurkina, Gyulnar Zhussupova, Aliya Smagulova, Venera Rakhmetova, Elmira Akhmedyarova, Aisha Darybayeva, Klara Kurmangaliyeva, Ilya Kukes

**Affiliations:** 1 Department of Internal Diseases, Astana Medical University, Astana 010000, Kazakhstan; zhussupbekova.l@amu.kz (L.Z.); rahmetova.v@amu.kz (V.R.); akhmedyarova.e@amu.kz (E.A.); darybayeva.a@amu.kz (A.D.); kurmangalieva.k@amu.kz (K.K.); 2 Heart Rhythm Research Institute, Astana Medical University, Astana 010000, Kazakhstan; gulnar.zh1@gmail.com; 3 Scientific Center for Clinical Metabolomics, Genetics, and Pharmacology, Moscow 109240, Russia; ilyakukes@gmail.com; 4 Scientific and Clinical Department of the International Association of Clinical Pharmacologists, Moscow 109240, Russia

**Keywords:** metabolomics, cardiovascular disease, myocardial infarction, metabolites, proteomics, multi-omics integration, biomarkers, personalized medicine, precision cardiology

## Abstract

Cardiovascular disease is a major cause of death worldwide and a global socio-economic problem. To date, there are numerous studies focused on finding new biomarkers of cardiovascular diseases. High-technological methods such as mass spectrometry (MS), high-performance liquid chromatography (HPLC), and nuclear magnetic resonance (NMR) spectroscopy enable us to record thousands of metabolites of organs and tissues. Studying organisms at a molecular level contributes to an in-depth understanding of preclinical conditions of various diseases. Metabolomics reflects the dynamics of metabolism distribution, including environmental influences, allowing us to create a metabolic profile of the patient. The aim of this review was to analyze current data on metabolomic and proteomic biomarkers in the diagnosis of cardiovascular diseases. The search databases were used to select studies on the potential clinical and diagnostic application of proteomic and metabolomic markers in cardiology. The selected sources were subjected to qualitative and thematic analysis. All biomarkers were grouped according to the pathophysiological process (inflammation, blood coagulation and lipid metabolism disorders, myocardial necrosis, etc.). The association of changes in metabolomic and proteomic profiles with the activation of pathogenic processes in the cardiovascular system was demonstrated. The use of these multivariate markers, individually or in combination, will increase the accuracy of early diagnosis and the effectiveness of treatment. This article also highlights the limitations of the method and possible ways to solve them.

## 1. Introduction

Cardiovascular diseases are the leading cause of morbidity and mortality worldwide. According to the World Health Organization, over the past three decades, the number of people suffering from cardiovascular disease has doubled and exceeded 500 million. Every year, these diseases cause the deaths of 17.9 million people worldwide (WHO) [1]. Kazakhstan is one of the regions with high cardiovascular morbidity and mortality [2]. According to the National Statistical Bureau, cardiovascular morbidity has also tended to increase over the past decade. Between 2010 and 2020, the incidence rate increased by 31% (from 2086.7 to 3024.4 per 100,000 population) [3]. Myocardial infarction and stroke are rightly considered the most life-threatening cardiovascular diseases. These pathologies are characterized by a high risk of sudden death and disability, which is associated with damage to vital organs—the heart and brain. In recent years, special attention has been paid to the growth of cardiovascular diseases among young people [4]. The increase in morbidity in this age group is associated with changes in lifestyle, increase in stress factors, improper nutrition, and low physical activity [5]. Early diagnosis of signs of ischemia, myocardial necrosis, or cerebrovascular disorders allows timely initiation of necessary treatment, reducing mortality. A comprehensive analysis of the pathogenesis of cardiovascular diseases requires an in-depth study of a set of risk factors, including hereditary predisposition, environmental influences, and individual metabolic features [6]. All these factors together determine the complexity of the nature of cardiovascular pathology, which requires an individual approach to diagnosis, prevention, and treatment.

To optimize cardiovascular disease management, the introduction of innovative diagnostic methods is very important. The integration of multi-omics approaches has attracted considerable interest in recent years. The combination of genomics, proteomics, lipidomics, and metabolomics allows for the accurate determination of individual risks and the development of personalized treatment and prevention strategies. The use of proteomics and metabolomics is particularly relevant for studying dynamic pathogenetic mechanisms of cardiovascular pathology. These methods panoramically reflect systemic metabolic processes in real time. The present review examines both classical (troponin and natriuretic peptides) and the most promising novel proteomic and metabolomic biomarkers—including tumor necrosis factor alpha, adhesion molecules ICAM-1 and VCAM-1, homocysteine, soluble ST2, methylarginine derivatives, and others—which collectively provide a holistic understanding of inflammation, remodeling, and metabolic disturbances in the cardiovascular system.

## 2. Methods

In order to obtain the most complete review of the use of multi-omics (proteomic/metabolomic) technologies in the diagnosis of cardiovascular diseases, an electronic search was conducted in the following search engines: PubMed, Google Scholar, Scopus, and Web of Science. Combinations of keywords and MeSH (Medical Subject Headings) terms were used to optimize the search. The following terms were included in the search in various combinations to ensure comprehensive coverage of publications: ‘metabolomic markers’, ‘metabolites’, ‘cardiovascular biomarkers’, ‘proteomics’, ‘omics technologies’, ‘cardiovascular disease’, and ‘myocardial infarction’. Original studies, qualitative reviews, and expert consensus studies on metabolomic and proteomic markers in the diagnosis of cardiovascular disease were included in the review. Studies including adult populations with sufficient data for interpretation, with a depth of about 10 years, were considered.

Current studies on cardiovascular biomarkers present several potential sources of bias, including heterogeneous patient populations, differences in sample collection and assay methods, lack of standardization, and multicenter validation. Publication bias may also exist due to preferential reporting of positive findings. These limitations should be considered when interpreting the diagnostic and prognostic value of biomarkers.

## 3. Results

### 3.1. New Approaches in Cardiology

**Figure 1:**
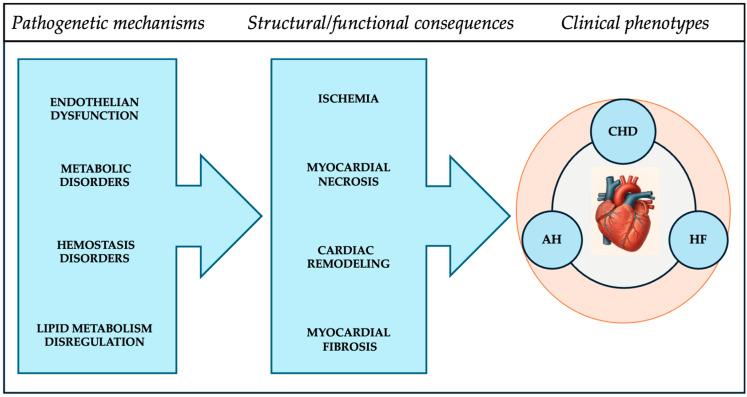
Systemic pathophysiological mechanisms and structural myocardial consequences in cardiovascular diseases (CHD—coronary heart disease; AH—arterial hypertension; HF—heart failure).

At the core of the pathogenesis of most cardiovascular diseases, such as coronary artery disease, arterial hypertension, and chronic heart failure, are common systemic processes: endothelial disfunction, lipid and metabolic disturbances, and hemostatic imbalance. These mechanisms lead to structural changes in the myocardium—remodeling, necroses, and fibrosis—which, in turn, cause progressive decline in systolic and diastolic cardiac function, and electrical instability. Collectively, these changes contribute to disease manifestation and aggravation of clinical course (Figure 1). An improved understanding of these mechanisms has prompted the development and exploration of novel diagnostic tools.

**Figure 2:**
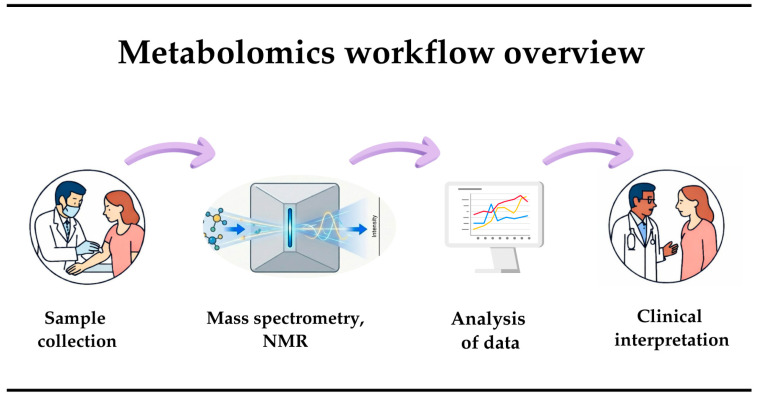
Overview of metabolomics workflow.

In recent years, cardiology has undergone a revolution due to the introduction of advanced technologies and innovative methods. Technologies such as artificial intelligence, big data analysis, and molecular imaging are being actively introduced into clinical practice, allowing doctors not only to detect diseases faster and more accurately, but also to predict their development. Automated systems allow for real-time monitoring of patients, which significantly increases the effectiveness of treatment. One of the most promising fields has become metabolomics, which studies the composition and dynamics of low-molecular-weight metabolites in the body. The introduction of modern technologies into clinical practice, such as metabolomics, improves the understanding of pathological processes before the actual phenotype of the disease and opens up opportunities for more accurate diagnostics. Metabolomics allows the study of low-molecular-weight metabolites in biological systems such as blood, urine, saliva, and tissues, and serves as a reflection of the functional activity of proteins and genes [7]. Unlike static genetics, metabolomics allows us to capture the dynamic activity of cells and the effects of environmental influences. The beginning of the metabolomics era is associated with the advent of nuclear magnetic resonance and gas chromatography in the 1960s and 1970s. The term itself was introduced in the late 1990s [8]. By analogy with genomics and proteomics, it denotes the study of all low-molecular-weight metabolites in a cell, tissue, or organism (Figure 2).

In recent years, there has been a rapid growth of interest in metabolomics in cardiology. According to the PubMed search engine, the number of studies on cardiovascular metabolomics increased more than tenfold from 2010 to 2019 [9]. This dynamic growth in publication activity indicates the growing relevance of research in this field.

All metabolite studies can be divided into targeted and non-targeted studies, with the possibility of identifying 100–200 or 1000–2000 metabolites simultaneously. Non-targeted studies provide the broadest possible metabolic footprint and are performed for broad early screening to identify novel pathogenic pathways and generate hypotheses. In contrast, targeted studies aim to identify specific, pre-selected metabolites. The choice of metabolites depends on the purpose and design of the study. At present, there is no single platform covering all metabolome profiles. However, several promising models are currently under active development [10]. There are metabolomic electronic databases such as HDMB [11] containing data on 220,945 low-molecular-weight metabolites. More than 700 of them are associated with cardiovascular diseases, with the main pathogenetic pathways being energy (Krebs cycle, β-oxidation), lipid metabolism (oxylipins, ceramides), amino acid metabolism (branched amino acids, arginine-NO system), microbiome-dependent pathways (Trimethylamine-N-oxide), and oxidative stress [12]. These pathways are reflected in Table 1.

The groups of metabolites listed in the table form a single metabolic network. Changes in one group lead to cascading shifts in others. Thus, BCAAs are inextricably linked to mitochondrial activity, acylcarnitines reflect the efficiency of β-oxidation, organic acids characterize the Krebs cycle activity, and lipid metabolites are associated with oxidative stress. It is the interconnection of these pathways that determines the systemic nature of metabolic disorders in cardiovascular diseases. Given the large amount of data in metabolomic studies, multilevel statistical approaches are required. Depending on whether the analysis is univariate or multivariate, adjustments are made. Machine learning is applied to identify the biological relevance of potential biomarkers. Such methods as Support Vector Machines, Random Forest, and eXtreme Gradient Boosting allow us to solve the problems of classification and selection of metabolites that may be markers of pathology [13]. In turn, not all machine learning methods are interpretable, which complicates their clinical application. To solve these problems, the most applicable method is Explainable Machine Learning (XAI)—a branch of artificial intelligence that allows the results of models to be obtained in an interpretable form. This is especially important for accurate diagnosis and treatment [14].

In modern clinical practice, “classical” biochemical markers are widely used, including troponin, creatine kinase-MB (CK-MB), myoglobin, alanine aminotransferase, aspartate aminotransferase, and natriuretic peptides (BNP and NT-proBNP) [15]. These indices make it possible to assess the degree of myocardial damage and impairment of its contractile function; each of them has a different degree of specificity and sensitivity. New data on the diagnostic role of previously known metabolomic markers have also been discovered. Special attention among them is paid to triglycerides [16], lipoprotein (a) [17,18], leucine [19,20,21], mTOR activity [22], dicarboxyacylcarnitines [23], and adipokines such as adiponectin and leptin [24,25]. The use of expanded biomarkers not only increases the accuracy of diagnosis of hidden disorders of carbohydrate and lipid metabolism but also helps to identify a patient’s predisposition to the development of diabetes, cardiovascular disease, and other metabolic diseases. All metabolomic markers, along with classical proteomic markers, can be classified according to pathophysiological processes (Table 2) [26].

Thus, some metabolites are involved in multiple pathogenetic pathways, acting independently or in combination with other metabolites. At early preclinical stages of cardiovascular pathology development, endothelial dysfunction, inflammation, and metabolic and neuroendocrine dysregulation with/without hemostasis disorders are more often observed. Subsequently, these pathogenetic mechanisms can lead to ischemia, injury, and necrosis, which have clinical manifestations. Diagnostic biomarkers of these clinical conditions are widely used in modern cardiology. Their use and interpretation are regulated by the recommendations of international professional societies. Developing myocardial and vascular wall fibrosis has no clear criteria, but its early detection and monitoring will prevent irreversible structural changes with subsequent heart failure.

In the following subsections, proteomic and metabolomic biomarkers will be discussed depending on the pathophysiologic processes.

### 3.2. Markers of Neuroendocrine Activation and Left Ventricular Function

Neuroendocrine activation is one of the key links in the pathogenesis of cardiovascular diseases. Since the discovery of this concept, which includes sympathoadrenal, renin–angiotensin–aldosterone, and natriuretic peptide systems, the interconnected network that accelerates the development of atherosclerosis has become evident. The imbalance between these systems determines the progression of most cardiovascular diseases. One of the markers of this dysregulation are natriuretic peptides, which are widely used in the diagnosis of heart failure. Being a part of the endogenous anti-stress system, they serve as biomarkers that reflect the degree of myocardial stretching and overloading, especially of the left ventricle, which allows us to objectively assess the presence and severity of heart failure [27]. Determination of their level in blood plasma not only helps to confirm the diagnosis but also allows us to monitor the patient’s condition in dynamics and predict the risk of adverse cardiovascular events. The study by J. B. Echouffo-Tcheugui et al. (2022), conducted within the prospective ARIC cohort and including 9789 individuals from the general population with and without previous cardiovascular disease, demonstrated the high prognostic value of NT-proBNP [28]. It was shown that individuals without prior cardiovascular history but with elevated NT-proBNP > 450 pg/mL had a risk of cardiovascular events—including all-cause mortality, atherosclerotic cardiovascular disease, and heart failure—which was comparable to that observed in patients with established diagnosis. In clinical practice, these peptides demonstrate high sensitivity and specificity, making them an invaluable tool for early diagnosis and risk stratification in patients with suspected acute or chronic heart failure.

The vasopressin system, including vasopressin and its receptors, plays an important role in maintaining body homeostasis and is considered an important diagnostic target. Vasopressin receptors (vascular, renal, and pituitary stress receptors) regulate systemic vascular resistance and circulating blood volume. Vasopressin enhances compensatory mechanisms aimed at maintaining perfusion. Given the low stability of vasopressin and technical difficulties in its determination, the copeptin-C-terminal part of the vasopressin prohormone is more often used in clinical diagnostics [29]. It is now considered as an important biomarker for identifying high-risk patients and assessing prognosis in a wide range of diseases. In a prospective cohort study by Davidson L. T. et al. (2025), it was demonstrated that elevated plasma copeptin levels (>5.6 pmol/L) are independently associated with an increased risk of major adverse cardiovascular events (MACE) in patients with type 2 diabetes [30]. Even though as a marker of neurohumoral stress, copeptin is not specific for cardiovascular disease, it was also indicated that the combined measurement of copeptin and troponin is particularly valuable in the diagnosis of myocardial infarction: their simultaneous evaluation achieves very high diagnostic accuracy and high negative predictive value in excluding acute myocardial infarction [31]. In addition, elevated copeptin levels are associated with a worse prognosis and a higher risk of complications after myocardial infarction [32].

One of the most sensitive markers of neurohumoral activation is adrenomedullin. Adrenomedullin (ADM) is a hormone produced in the endothelium and vascular smooth muscle, which maintains the endothelial barrier under volume overload [33]. Its stable fragment reflects the severity of endothelial dysfunction, microcirculatory changes, and overload stress. In addition, ADM affects the renin–angiotensin system by inhibiting it. Elevated ADM levels in patients with acute heart failure are associated with congestion and clinical outcomes resulting from these pathologic processes [34]. In a multicenter prospective observational study, it was shown that elevated levels (>29 pg/mL) of bio-adrenomedullin (bio-ADM) in patients with cardiac amyloidosis are associated with a higher incidence of MACE. Bio-ADM was an independent predictor of outcomes after adjustment for age and sex [35].

Another marker of neurohumoral system activation is Endothelin-1, a potent vasoconstrictor. It also contributes to processes such as inflammation and proliferation in the vessel wall, increased thrombosis, impaired myocardial contractile function, and cardiac arrhythmias [36]. Endothelin-1 affects systemic hypertension by playing an important role in sodium excretion by the kidneys [37]. It was recently found that intravenous administration of Big ET-1, a precursor of endothelin-1, leads to an increase in urinary sodium excretion (reflected in an increase in fractional sodium excretion), as well as an increase in free water clearance, presumably due to the conversion of Big ET-1 to the active form of ET-1 [38]. Other studies have demonstrated that detection of ET-1-related peptides may be useful for predicting cardiovascular events in patients with stable coronary heart disease. Yang C et al. demonstrated that elevated plasma ET-1 concentrations have independent prognostic value for assessing outcomes in patients with coronary heart disease, including such factors as erythrocyte sedimentation rate and fibrinogen levels. ET-1 levels have been investigated as risk predictors for acute myocardial infarction [39]. Several studies have shown that ET-1-related peptide levels were associated with a greater risk of adverse events after myocardial infarction [40]. The Leicester Acute Myocardial Infarction Peptide (LAMP) study found that the level of C-terminal pro-ET-1 was an independent predictor of death and/or heart failure in patients after acute myocardial infarction [41].

### 3.3. Inflammatory Markers

In the context of modern understanding of the pathogenesis of atherosclerosis, particular attention is paid to the role of inflammation as a universal mechanism of vascular wall damage. A number of studies emphasize the key role of inflammatory markers in predicting cardiovascular disease [42,43,44]. For example, in the EPIC-Norfolk study, combined indicators (lipoprotein (a) and LDL) together with high-sensitivity C-protein predicted the risk of MACE (major adverse cardiac events) [45]. Similar conclusions were made in meta-analyses, which noted that elevated levels of C-reactive protein are associated with an increased risk of myocardial infarction and other complications [46]. A review by Mantovani A et al. (2023) emphasizes the role of acute-phase proteins in assessing prognosis in patients with cardiovascular disease [47].

Other key mediators of the inflammatory response are interleukin-6, tumor necrosis factor, and endothelial adhesion molecules. Geovanni GR and colleagues (2018) thoroughly studied the contribution of inflammatory cytokines, such as interleukins and tumor necrosis factor alpha (TNF-α), as well as ICAM-1 and VCAM-1 adhesion molecules, in the pathogenesis of atherosclerosis and found that their elevation is associated with disease progression and atherosclerotic plaque instability [48]. Koenig and Khuseinova also noted the importance of biomarkers such as YKL-40, CD40L, and other interleukins, identifying their role in plaque instability and the assessment of cardiovascular complication risk [49]. A study by O’Donoghue and colleagues showed that the level of growth and differentiation factor GDF-15, which suppresses macrophage response and proteolytic enzyme synthesis, serves as an independent predictor of adverse outcomes in patients with acute coronary syndromes [50]. The adhesion molecules ICAM-1, VCAM-1, and ELAM-1 play a key role in the pathogenesis of atherosclerosis by promoting inflammation and damage to the vascular wall. They cause the adhesion and migration of leukocytes, such as monocytes and lymphocytes, into the vessel wall, which is the first step in the development of atherosclerotic plaques [51]. Jiancai Yu and colleagues (2022) also found in their studies that the adhesion molecules ICAM-1, VCAM-1, and ELAM-1 play an important role in predicting adverse outcomes in patients with ischemic heart disease. However, VCAM-1 remained an independent predictor [52].

It is known that TNF-α has a significant effect on the metabolism of lipids, fats, carbohydrates, and minerals. It regulates cholesterol synthesis processes and promotes the formation of atherogenic lipid fractions and apolipoproteins in the liver [53]. At the same time, TNF-α slows down the breakdown of cholesterol and its excretion with bile acids, and also increases the production of triglycerides [54]. In addition, TNF-α plays an important role in initiating and maintaining inflammatory reactions in the vascular wall. In diseases such as atherosclerosis, ischemic heart disease, and arterial hypertension, there is increased production of TNF-α and enhanced expression of its receptors [55].

The transmembrane protein NOTCH1 plays a central role in signaling pathways that regulate the development and specialization of vascular wall cells [56]. Several studies emphasize that changes in NOTCH1 gene expression and mutations are associated with an increased risk of atherosclerosis, heart valve calcification, and various vascular diseases [57,58]. For example, the review article “The Role of the Notch Signaling Pathway in Endothelial Progenitar Cell Biology” (Kwon et al., Trends in Cardiovascular Medicine, 2009) describes in detail the involvement of NOTCH1 in inflammatory processes, changes in vascular structure, and the formation of unstable atherosclerotic plaques [59].

Interferon-gamma (IFNγ), a cytokine whose elevated levels are associated with the progression of atherosclerosis, plays a similar role in plaque destabilization [60]. A review by Ailin Elyasi (2020) showed that IFNγ promotes macrophage activation and enhances the inflammatory process in the vessel wall, thereby increasing the risk of cardiovascular complications [61]. All of the markers mentioned—NOTCH1, GDF-15, and IFNγ—are being actively studied as biomarkers and potential therapeutic targets in the context of cardiovascular disease [62,63,64]. Their levels in the blood reflect the degree of inflammation, damage to the vascular wall, and the risk of adverse outcomes, as confirmed by numerous scientific publications. In a study by Maisel A. et al., markers of inflammation and stress, such as ST2 (suppression of tumorigenicity 2), were analyzed for their use in predicting cardiovascular disease, particularly acute heart failure. The researchers found that this marker plays a detrimental role in the pathogenesis of chronic inflammatory cardiovascular diseases [65,66].

### 3.4. Hemostasis System Markers (Coagulation Factors)

Modern cardiology pays special attention to the search for biomarkers that not only reflect the state of the coagulation and inflammatory systems but also allow for the prediction of cardiovascular events. Homocysteine, a sulfur-containing amino acid, is an intermediate in the normal biosynthesis of the methionine and cysteine, and plays a key role in the methylation cycle [67]. There is substantial evidence on the causes and consequences of hyperhomocysteinemia [68,69]. The main genetic cause of severe hyperhomocysteinemia and classic homocystinuria is homozygous CβS deficiency [70]. Hyperhomocysteinemia can also develop due to MTHFR deficiency, methionine synthase deficiency, vitamin B12 metabolism disorders, and folate, vitamin B6, and vitamin B12 deficiency [71]. The prevalence of coronary heart disease, as well as carotid and peripheral vascular disease, strongly correlates with total serum homocysteine levels, as confirmed by several cross-sectional and case–control studies [72,73]. Homocysteine is recognized as an independent risk factor for atherosclerosis. In a systematic review and meta-analysis by Unadka S.V. et al. (2024) including 59 studies with 9381 patients with CAD and 12,188 controls, circulating homocysteine levels were higher in patients with CAD compared with control populations (SMD of 0.73, 95% CI: 0.55–0.91) [74]. The ability of homocysteine to affect endothelial cells and vascular smooth muscle cells causes cardiovascular disorders, including structural and functional changes in arteries at the preclinical stage [75]. An umbrella review including 135 observational meta-analyses, 106 Mendelian randomization studies, and 26 interventional meta-analyses showed that elevated homocysteine is casually associated with stroke. Homocysteine lowering therapy significantly reduced stroke risk [76].

Among other well-known key markers of inflammation and blood clotting, fibrinogen occupies a special position. Elevated fibrinogen levels are significantly associated with the risk of heart attack and stroke [77,78]. Thus, an article by Surma S and Banach M (2021) presents a review of current data on the role of fibrinogen in the development of atherosclerotic cardiovascular diseases [79]. The authors emphasize the importance of fibrinogen as a biomarker and as a potential therapeutic target in the prevention of cardiovascular events. In another study (S. G. Thompson et al.), involving 3000 patients with angiographically confirmed CHD, fibrinogen, rather than cholesterol, was identified as an independent risk factor for coronary events. The risk of coronary events increased with rising plasma fibrinogen concentrations and remained low with high cholesterol levels until fibrinogen concentrations became low. Fibrinogen concentrations were on average 6.5% higher in the group with coronary events than in the group without events (*p* = 0.01) [80]. Fibrinopeptide A (FPA) is also involved in the pathogenesis of cardiovascular diseases, indicating increased activity of the blood coagulation system. Elevated FPA levels have been observed in patients with acute coronary syndrome, myocardial infarction, and sudden coronary death, highlighting its role in thrombus formation [81]. Recent research has highlighted the role of the membrane adhesion protein P-selectin, which is localized in platelet alpha-granules and initiates the thromboinflammatory response. Hidenori Koyama et al. noted that, compared with healthy control groups, patients with higher expression of P-selectin, which is the main ligand of PSGL-1, showed an association with carotid atherosclerosis [82]. The percentage of P-selectin-positive platelets was significantly higher in subjects with carotid plaque (median 1.72) than in those without plaque (median 0.80; *p* < 0.0001). This suggests that PSGL-1 expression may be associated with cholesterol levels in addition to its role in the pathogenesis of hypertension.

Among the markers of the hemostatic system, tissue plasminogen activator (tPA) has been actively studied since the 1990s. In recent years, researchers have focused on the role of plasminogen activator inhibitor (PAI-1). A review by Alireza Khoddam (2025) discusses the role of plasminogen activator inhibitor (PAI-1) in the aging of the cardiovascular system, with a particular focus on cellular aging [83]. Increased PAI-1 expression accelerates the development of age-related pathologies: it increases vascular stiffness through collagen deposition and reduced nitric oxide bioavailability, and accelerates cellular aging through the SASP phenotype and IGFBP-3 signaling pathway [84]. Thus, PAI-1 demonstrates the link between cardiovascular pathology and inflammation, or “inflammatory aging.”

One of the key factors in platelet adhesion and aggregation is von Willebrand factor (VWF) [85]. This is particularly relevant in areas with high blood flow velocity, such as coronary arteries with stenotic or ruptured atherosclerotic plaques [86]. Elevated plasma VWF levels have been shown to be associated with established traditional risk factors for cardiovascular disease, as well as with the likelihood of adverse events [87]. In the study of van Paridon P.C.S. et al. (2022) [88], one of the main observations was that vWF activity below 76% (corresponding to the lowest quintile of the reference population) was associated with a 40% reduction in cardiovascular disease risk, independent of age, sex, traditional risk factors, and prior CVD. In addition, vWF antigen levels below 83% were linked to a 40% lower risk of all-cause mortality [89].

The contemporary literature pays particular attention to markers of endothelial damage, in particular thrombomodulin (sTM) [90]. Thrombomodulin is a transmembrane glycoprotein expressed on the surface of endothelial cells that regulates blood coagulation by binding to thrombin and activating protein C [91]. Endothelial damage associated with inflammatory and atherosclerotic processes leads to a decrease in thrombomodulin expression and its release into the bloodstream in soluble form. A number of scientific studies indicate that elevated concentrations of soluble thrombomodulin in blood plasma are associated with a higher risk of adverse cardiovascular events, such as myocardial infarction, stroke, and acute heart failure [92,93]. In the study by Misirlioglu, N.F (2025), serum levels of thrombomodulin, H_FABP, pentraxin-3, and galectin-3 were significantly higher in patients with STEMI and NSTEMI compared with controls (*p* < 0.001) and showed positive correlations with NT-proBNP [94]. Esmon C.T. et al. (2014) emphasize that sTM can not only serve as a marker of endothelial damage but also reflect the severity of inflammation in acute coronary pathology [95]. Some studies have noted that monitoring sTM levels in patients with coronary artery disease can improve risk stratification and aid in the early detection of vascular disease progression [96]. Thus, thrombomodulin is considered a promising biomarker for assessing the condition of the vascular wall and predicting cardiovascular disease, as well as a potential therapeutic target in endothelial protection strategies.

### 3.5. Predictors of Lipid Metabolism Disorders

The link between lipids and the risk of cardiovascular disease has been confirmed by numerous clinical and epidemiological studies. One of the most famous is the Framingham study, which began in 1948 and showed that elevated levels of total cholesterol and lipoproteins (LDL) significantly increase the risk of coronary heart disease, myocardial infarction, and other cardiovascular diseases [97]. At the same time, high levels of high-density lipoprotein (HDL) are associated with a protective effect that reduces the likelihood of developing atherosclerosis and related complications. Large meta-analyses, such as INTERHEART and MESA, have confirmed that apolipoprotein B (the main protein of LDL and VLDL) is also one of the key prognostic biomarkers of cardiovascular risk, and its elevation is directly associated with the progression of atherosclerotic changes in blood vessels [98]. On the other hand, apolipoprotein A1 (the main protein of HDL) has antiatherogenic properties, promoting the removal of cholesterol from the vascular wall [99]. Thus, monitoring the lipid profile, including total cholesterol, LDL, HDL, and apolipoproteins, is an important part of risk assessment and prevention of cardiovascular complications [100].

Retinol-binding protein 4 (RBP4) is a transport protein for retinol (vitamin A) in the blood and is considered a promising marker of metabolic disorders, particularly insulin resistance and the likelihood of developing cardiovascular disease [101]. Some studies have shown that high levels of RBP4 are associated with impaired glucose metabolism and an increased risk of atherosclerosis and type 2 diabetes [102]. For example, a study by Graham TE et al. (2006) demonstrated that plasma RBP4 concentration correlates with insulin resistance in patients with obesity and type 2 diabetes [103].

A prospective study by Sun Q et al. (2013) confirmed the association between RBP4 levels and the risk of cardiovascular events, especially in patients with metabolic syndrome [104]. Leptin, a hormone produced by adipose tissue, regulates appetite and energy metabolism and is also considered a prognostic marker for cardiovascular disease. Elevated leptin levels are associated with an increased risk of coronary heart disease, hypertension, and stroke [105]. A study by Wallace AM et al. (2000) found that patients with higher leptin levels were more likely to show signs of atherosclerosis and hypertension [106]. A meta-analysis conducted by Martin SS et al. (2014) demonstrated that leptin is an independent predictor of cardiovascular events, especially in individuals with obesity and metabolic syndrome [107]. In addition, a study by N Hansen et al. (2025) found a direct link between leptin concentration and the risk of myocardial infarction [108].

### 3.6. Myocardial Fibrosis Markers

Myocardial fibrosis is a serious complication of both acute and chronic heart disease, contributing to an overall increase in cardiovascular risk and increasing the likelihood of sudden cardiac death. Although most structural changes in the heart are irreversible, early detection of this pathology can help reduce the severity of symptoms and improve the long-term prognosis for patients. The most actively studied biomarkers of myocardial fibrosis and cardiac tissue remodeling include compounds such as galectin-3, tissue inhibitors of matrix metalloproteinases (TIMP-1 and TIMP-2), type I procollagen propeptide (PICP), matrix metalloproteinases (MMP-9 and MMP-3), ST2 growth factor, NT-proBNP, and collagen IV. In addition, lipid metabolism indicators (total cholesterol, low- and high-density lipoproteins, and triglycerides) and extracellular matrix components are also associated with fibrosis.

One of the important mediators of cardiac fibrosis is galectin-3, a lectin involved in intercellular interactions and the regulation of inflammatory responses [109]. Elevated levels of galectin-3 in blood plasma are associated with the development and progression of cardiac fibrosis, as well as with an unfavorable prognosis in patients with chronic heart failure [110]. Sherpa M.D. et al. (2023) [111] found that increasing galectin-3 levels were associated with cardiac fibrosis. Compared to non-SCD cases, subjects with SCD had significantly greater myocardial fibrosis involving both the left ventricular free wall and septum. The mean fibrosis area was 25.0 ± 4.09 in animals with SCD versus 12.01 ± 1.8 in controls (*p* = 0.0361).

Galectin-3 enhances fibroblast activation and extracellular matrix component synthesis, increasing myocardial stiffness and impairing myocardial function [111].

Tissue inhibitors of matrix metalloproteinases—TIMP-1 and TIMP-2—control the activity of matrix metalloproteinases, which are responsible for the breakdown of collagen and other proteins in the extracellular matrix [112]. An imbalance between matrix metalloproteinases and their inhibitors leads to excessive collagen deposition, fibrosis, and myocardial remodeling [113]. PICP (procollagen type I propeptide) and collagen IV reflect the activity of synthesis and deposition of new collagen fibers in the myocardium, serving as direct markers of fibrosis formation. Elevated levels of these markers in the blood indicate progressive fibrosis, which is important for early diagnosis and assessment of treatment effectiveness [114].

Another marker associated with cardiac stress and inflammation is the growth factor ST2 (especially its soluble form, sST2). High ST2 levels are associated with an increased risk of heart failure and adverse outcomes, which is explained by its role in myocardial remodeling and fibrosis [115]. NT-proBNP may also indirectly indicate the severity of fibrotic changes, as increased cardiac wall stiffness impedes effective relaxation and ventricular filling [116]. Lipid metabolism indicators (total cholesterol, LDL, HDL, and triglycerides) are not direct markers of fibrosis, but their impairment contributes to the progression of atherosclerosis, ischemia, and the secondary development of fibrotic changes in the myocardium [117].

Thus, comprehensive analysis of these biomarkers allows us not only to determine the presence and degree of cardiac fibrosis but also to assess the risk of cardiovascular complications and select individual treatment approaches aimed at slowing down the progression of the pathological process.

### 3.7. Myocardial Necrosis Markers

In recent years, myocardial necrosis markers such as creatine kinase (CK) and its MB fraction, troponins, myoglobin, lactate dehydrogenase (LDH), and aspartate aminotransferase (AST) have been the focus of active clinical research [118,119]. Particular attention is paid to troponins, which are recognized as the most specific and sensitive biomarkers of heart muscle damage. Various studies have shown that elevated troponin levels in the blood correlate with the severity of ischemic damage and serve as a criterion for diagnosing myocardial infarction, as well as for risk stratification in patients with acute coronary syndromes [120]. In addition, analysis of the dynamics of CK-MB and myoglobin levels allows the time of necrosis onset to be estimated and the effectiveness of reperfusion therapy to be monitored [121]. In recent years, work has been underway to identify new markers that could improve diagnostic accuracy; for example, studies of cardiac-specific microRNAs are being conducted [122]. Zhang (2025) also demonstrated a link between metabolites such as ceramides (Cer(d18:1/16:0)) and acute coronary syndromes [123]. These studies are aimed at the earlier detection of myocardial necrosis, the prediction of complications, and the individualization of treatment for patients with acute cardiovascular events [124].

### 3.8. Markers of Endothelial Dysfunction

Endothelial dysfunction is a universal mechanism in the development of cardiovascular diseases and manifests itself in a change in the balance between the synthesis of factors promoting vasodilation, the protection of the vascular wall, and the inhibition of cell proliferation, and the production of substances that cause vasoconstriction, increase the tendency to thrombosis, and enhance cell proliferation [125]. In modern clinical practice, substances such as homocysteine, nitric oxide (NO), endothelin 1–21, endothelial NO synthase (eNOS), asymmetric dimethylarginine (ADMA), endoglin, endocan, as well as intercellular adhesion molecule (ICAM-1) and vascular cell adhesion molecule (VCAM-1), have been identified as relevant biomarkers of endothelial dysfunction. Nitric oxide, synthesized with the participation of NO synthase enzymes, plays a key role in regulating smooth muscle contractions, providing vasodilation under normal conditions [126]. However, due to its short half-life, its determination in the body presents certain difficulties [127].

Endothelin-1 is a potent vasoconstrictor associated with more severe disease progression and poor prognosis [128]. However, the widespread use of this marker is limited by the difficulty of obtaining accurate data due to constant fluctuations in blood flow velocity. S- and L-endoglin (s-l-ENG) are transmembrane glycoproteins that can be used as biomarkers for various cardiovascular and metabolic disorders, such as preeclampsia, hypercholesterolemia, familial hypercholesterolemia, atherosclerosis, and hypertension [129,130]. For example, in a study by J. Urbankova Rathouska (2025), patients with myocardial infarction had significantly elevated levels of sENG and endocan in plasma compared to the control group [131].

Asymmetric dimethylarginine (ADMA) is an endogenous inhibitor of nitric oxide (NO) synthesis, a key vasoactive mediator [132]. An increase in ADMA concentration leads to a decrease in NO production, which causes endothelial dysfunction and vasoconstriction, and contributes to the progression of hypertension and atherosclerosis. In a study by Janes, F. (2019), patients were found to have significantly higher levels of ADMA in their blood plasma compared to the control group [133]. High ADMA levels are associated with a negative prognosis in patients with cardiovascular disease [134]. Intercellular adhesion molecule-1 (ICAM-1) and vascular cell adhesion molecule-1 (VCAM-1) belong to the immunoglobulin family, which is involved in the attachment of leukocytes to endothelial cells during acute and chronic inflammatory processes [135].

ICAM-1 is responsible for the migration of leukocytes to the site of inflammation [136]. VCAM-1 is another inflammatory cell adhesion molecule that regulates vascular inflammatory adhesion and transendothelial migration of leukocytes [137]. The initial stage of atherosclerosis is caused by endothelial dysfunction, which is associated with overexpression of ICAM-1 and VCAM-1 [138]. Several studies have also found that acute myocardial infarction and other coronary syndromes are associated with ICAM-1 and VCAM-1. Lino et al. (2019) demonstrated significantly elevated levels of ICAM-1 and VCAM-1 adhesion molecules in individuals with heart failure [139].

### 3.9. Markers of Metabolic Disorders

In recent years, markers of metabolic disorders have become increasingly important in the assessment of risk and diagnosis of cardiovascular diseases such as coronary heart disease (CHD), myocardial infarction, and heart failure. Particular attention is paid to branched-chain amino acids (BCAAs) and their derivatives—branched-chain α-keto acids (BCKAs)—as well as indicators of mTOR signaling pathway activity, leucine levels, triglycerides, and dicarboxyacylcarnitines. S. S. Markin and his colleagues found that patients with IHD have impaired homeostasis of BCAAs and their derivatives (BCKAs), which complements traditional biochemical markers such as troponin, creatine kinase, myoglobin, and natriuretic peptides [140]. The authors note that BCAA homeostasis disruption and accumulation led to mTOR signaling pathway hyperactivation, energy metabolism disorders, insulin resistance, and inflammation. This contributes to the development of coronary heart disease, diabetes, obesity, and accelerated aging. A new approach using a metabolomic panel includes the assessment of BCAAs, BCKAs, dicarboxyacylcarnitines, and natriuretic peptides and troponins, which improves the accuracy of diagnosis and understanding of the mechanisms underlying coronary artery disease [141]. Shah et al. (2012), in a study of 2023 patients who underwent selective cardiac catheterization, demonstrated a link between long-chain dicarboxyacylcarnitines, medium-chain acylcarnitines, branched-chain amino acids, and fatty acids and death or myocardial infarction [142].

Thus, studying the profile of branched-chain amino acids (BCAAs—valine, leucine, and isoleucine) and their derivatives, such as acylcarnitines and succinate, allows us to reflect the degree of metabolic disorders in cardiovascular diseases. A number of studies have noted that an increase in the concentration of certain amino acids and their derivatives correlates with the risk of developing heart failure, ischemia, and an unfavorable prognosis.

## 4. Discussion

Cardiovascular diseases are the leading cause of non-communicable diseases and rank first in terms of morbidity and mortality [143]. In this regard, early diagnosis of cardiovascular diseases is the focus of attention of scientists around the world. This is particularly important for the younger cohort, as the increase in morbidity in this population is associated with socio-economic consequences [144]. In this regard, there is a growing need for new technologies capable of detecting hidden pathophysiological changes.

A problem in modern clinical practice is a certain lack of objective laboratory tests that allow pathological processes to be diagnosed at stages preceding clinical manifestations. Existing highly sensitive biological markers of cardiovascular disease, such as troponin and natriuretic peptides, reflect processes in the late stages and are not strictly specific. There is a need for markers that would reflect multifactorial pathological processes in the cardiovascular system at their minimum severity.

The introduction of high-tech omics methods can solve this problem and significantly expand the possibilities for precision medicine. Metabolomics will enable the creation of individual profiles reflecting biochemical reactions in response to environmental factors and each patient’s lifestyle. The identified changes will not only allow for the diagnosis of pathological processes at the preclinical stage but also enable appropriate interventions to be carried out, which will improve primary prevention of cardiovascular diseases.

This review describes the possibilities for diagnosing key pathophysiological processes that trigger most cardiovascular diseases: endothelial dysfunction, chronic inflammation of the vascular wall, oxidative stress, and activation of neurohumoral systems (RAAS and sympathoadrenal). Modern biomarkers such as asymmetric dimethylarginine, adhesion molecules (ICAM-1 and VCAM-1), branched-chain amino acids and their derivatives, adrenomedullin, homocysteine, and other indicators play a key role in these processes.

Chronic inflammation is accompanied by increased levels of homocysteine, inflammatory cytokines, and cell adhesion molecules, contributing to the development of atherosclerosis. Activation of neurohumoral systems leads to vasoconstriction, fluid retention, and vascular remodeling. At the same time, natriuretic peptides, which reflect the stage and severity of heart failure, are not sufficiently informative in the early stages when the functional capacity of the heart is still preserved. Endothelial dysfunction, which underlies the development of myocardial remodeling, is accompanied by an imbalance in the nitrergic system, the state of which we can indirectly judge by the derivatives of asymmetric dimethylarginine.

Metabolic disorders manifest themselves both through an imbalance of certain amino acids and their derivatives and through changes in energy metabolism, being closely linked to the mechanisms of oxidative stress. Thus, changes in the metabolite profile reflect not only the degree of damage to the myocardium and vascular wall, but also the cascade of processes preceding the clinical manifestation of the disease. Comprehensive analysis of biomarkers enables differentiated management of patients at the earliest stages of cardiovascular disease and minimizes adverse outcomes at the preclinical stage.

However, despite the positive prospects for the application of omics methods in clinical practice, they are quite complex due to their high dimensionality, variability in metabolite concentrations, high cost of research, the need to separate/purify the sample before loading it into a mass spectrometer, and insufficient analytical sensitivity at low metabolite concentrations. Another problem is the lack of uniform standards for conducting metabolomic studies and interpreting their results, which makes it difficult to compare data obtained in different medical centers and among different population groups [133]. Many potential markers require further validation in larger patient groups to confirm their clinical significance and reproducibility. In addition, the long-term prognostic value of most metabolomic markers remains insufficiently studied, which limits their widespread use in clinical practice. The complexity of interpreting the data obtained is also exacerbated by the high variability of metabolic profiles, which may depend not only on pathology but also on age, ethnicity, gender, nutrition, comorbidities, and drug therapy. This high variability limits the comparability and generalizability of findings across the studies. Therefore, the interpretation of metabolomic biomarkers requires caution, and future research should incorporate careful population stratification and systematic control of potential confounding factors to enhance reproducibility and clinical relevance.

In 2023, the American Heart Association (AHA) issued a release summarizing the practical use of metabolomics in cardiology, outlining key approaches to incorporating metabolomic markers into the diagnosis and risk stratification of cardiovascular disease [145]. The association recommends integrating metabolomic data with traditional clinical and laboratory indicators to create multifactorial risk assessment models, which, in their opinion, will increase the accuracy of diagnosis and improve treatment outcomes. In addition, this document notes the limitations of the method’s application in cardiology.

Thus, the widespread introduction of new markers into routine practice requires further large-scale studies aimed at standardizing methods and assessing their prognostic significance. However, the need to continue developing metabolomics, despite the existing difficulties, lies in the fact that this innovative approach opens up new horizons for early diagnosis, risk stratification, and individualization of therapy for cardiovascular diseases.

## 5. Conclusions

The use of metabolomic and proteomic markers allows us to identify hidden mechanisms of pathogenesis, predict undesirable events, and select the most appropriate therapeutic approaches for each individual patient. This is particularly important given the high heterogeneity of clinical manifestations and the unique characteristics of patients with cardiovascular diseases. Their integration into clinical practice will not only allow for a more accurate assessment of the risk of complications but will also provide a more complete understanding of the molecular mechanisms underlying atherosclerosis, ischemic heart disease, and heart failure. The use of comprehensive proteomic and metabolomic panels in diagnostics contributes to the personalization of treatment approaches and opens up new opportunities for the development of targeted therapeutic strategies aimed at correcting metabolic disorders.

Thus, the introduction of multi-omics panels contributes to the development of personalized medicine, where therapy is selected according to unique metabolic profiles, which increases the effectiveness of treatment and reduces the risk of complications. Continued progress in this area is an important step toward personalized medicine and improving the effectiveness of prevention and treatment of cardiovascular diseases.

## 6. Future Directions

In the future, the field of metabolomics in cardiology is expected to grow significantly, driven by continuous technological innovations and a deeper understanding of metabolic processes. The development of highly sensitive analytical methods, such as high-resolution mass spectrometry and advanced bioinformatics tools, is expected to improve the detection and interpretation of subtle metabolic changes associated with the onset and progression of cardiovascular disease. One promising avenue is the integration of metabolomic data with other omics disciplines—such as genomics, proteomics, and transcriptomics—to generate comprehensive molecular profiles. This multilevel approach can reveal complex interactions between genes, proteins, and metabolites, which will allow for a better understanding of the complex mechanisms underlying cardiovascular pathology and identify new therapeutic targets. In addition, future research should focus on validating metabolomic biomarkers in different populations and clinical settings to ensure their reliability and relevance for everyday use. Large-scale multicenter studies will be necessary to establish reliable reference ranges and assess the prognostic value of new markers in real-world patient cohorts. Work on standardizing protocols for sample collection, processing, and analysis must continue, as these will form the basis for reproducibility and comparability of results across different laboratories and healthcare systems. Artificial intelligence technologies will make a significant contribution to achieving reliability and standardization of results. Collaboration between academic researchers, clinicians, and industry partners will be crucial for translating scientific advances into practical diagnostic tools and personalized treatment strategies. Finally, ongoing training for healthcare professionals will help bridge the gap between scientific research and clinical practice, enabling them to effectively interpret metabolomic data and apply it to patient care. As the cost of technology decreases and expertise increases, metabolomic panels are likely to become an integral part of cardiovascular disease diagnosis and risk management, contributing to more accurate and patient-centered medical care.

## Figures and Tables

**Table 1:** Metabolites associated with cardiovascular diseases (according to PUBMED and SCOPUS).

Group of Metabolites	Examples	Pathophysiological Role	Pathways	Type of MS	Number of Studies
Amino acids (BCAAs)	Leucine, Isoleucine, Valine	Insulin resistance, mTOR, remodeling	Protein metabolism, NO pathway	LC-MS/MS	≈450
Acylcarnitines (Acs)	C2, C8, C14:1	Mitochondrial dysfunction	β-oxidation	LC-MS/MS	≈320
Lipids/Phospholipids	LPC, PE, SM	Atherogenesis, inflammation	Lipid metabolism	LC-MS/MS	≈700
Sphingolipids/Ceramides	Cer(d18:1/16:0)	MACE risk, endothelium	Sphingosine pathway	LC-MS/MS	≈280
Oxylipins	12-HETE, 15-HETE	Inflammation, thrombosis	LOX/COX	LC-MS/MS	≈260
Amino acid derivatives	TMAO, ADMA, SDMA	NO-dysfunction, microbiome	Arginine-NO pathway	LC-MS/MS	≈600
Organic acids	Lactate, Succinate	Ischemia, hypoxia	Krebs cycle	GC-MS	≈340
Purines	Adenosine, Inosine	Ischemia/reperfusion	Purine metabolism	LC-MS/MS	≈210

**Table 2:** Metabolomic markers of pathophysiological processes (O. V. Astafyeva et al. [26] with additions by the authors).

Group №	Title	Examples/Laboratory Biomarkers of Cardiovascular Diseases
I	Markers of left ventricular function and neuroendocrine activation	Natriuretic peptides/natriuretic hormones *, brain natriuretic peptide (BNP, or NT-proBNP) *, atrial natriuretic peptide (ANP) *, Cardiac troponins (hs-cTnT, or troponin T; hs-cTnI, or troponin I) * Copeptin ** Adrenomedullin ** Endothelin-1 ** Melatonin **
II	Inflammatory markers	Adhesion molecules ICAM-1, VCAM-1, ELAM-1 ** Interleukin-1α, -1β, -4, -5, -6, -8, -10, -12, -13, -17, -18, etc. ** Tumor necrosis factor (TNFα) ** Cartilage glycoprotein 39 (YKL-40) ** Soluble CD 40 ligand (sCD40L) ** Transmembrane protein NOTCH1 ** Growth differentiation factor 15 (GDF-15) ** Suppression of tumorigenicity 2 (ST-2) ** Interferon gamma (IFNγ) ** Lipoprotein-associated phospholipase **
III	Hemostasis system markers (coagulation factors)	Fibrinopeptide A ** P-selectin ** Tissue plasminogen activator (t-PA) ** Fibrinogen * Homocysteine * Von Willebrand factor (VWF) * Endothelin ** Thrombomodulin **
IV	Predictors of lipid metabolism disorders	Total cholesterol * Very low-, low-, and high-density lipoproteins * Apolipoprotein A1, Apolipoprotein B * Triglycerides * Retinol-binding protein type 4 ** Leptin ** Homocysteine *
V	Markers of myocardial fibrosis	Galectin-3 ** Tissue inhibitor of matrix metalloproteinases (TIMP-1, -2) ** PICP ** Matrix metalloproteinases (MMP-9, -3) ** Growth stimulating factor (ST2) ** NT-proBNP * Total cholesterol * Low- and high-density lipoproteins * Triglycerides * Collagen IV **
VI	Myocardial necrosis markers	Cardiac troponins (hs-cTnT, or troponin T; hs-cTnI, or troponin I) * Creatine phosphokinase (MB fraction) * Myoglobin * Lactate dehydrogenase (LDH) * Aspartate aminotransferase (AST) * ceramides (Cer(d18:1/16:0)) **
VII	Markers of endothelial dysfunction	Homocysteine* Asymmetric dimethylarginine (ADMA) ** Endothelin 1–21 ** Intercellular adhesion molecule 1 (ICAM-1) ** Vascular cell adhesion molecule 1 (VCAM-1) ** Endothelial nitric oxide synthase (eNOS) **
VIII	Markers of metabolic disorders	Branched-chain amino acids (BCAA) ** Branched-chain α-keto acids (BCKA) ** Leucine ** mTOR activity indicators ** Triglycerides * Dicarboxyacylcarnitines **

Note: * classical biomarkers used in practical medicine; ** new biomarkers with diagnostic potential, currently under study.

## Data Availability

Data sharing does not apply to this article, as no datasets were generated or analyzed during the current study.

## Abbreviations

The following abbreviations are used in this manuscript:

ADMA	Asymmetric dimethylarginine
ADM	Adrenomedullin
AHA	American Heart Association
ALT	Alanine aminotransferase
ANP	Atrial natriuretic peptide
Apo-B	Apolipoprotein B
AST	Aspartate aminotransferase
BCAAs	Branched-chain amino acids
BCKAs	Branched-chain α-keto acids
BNP	Brain natriuretic peptide
CPK	Creatine phosphokinase
CHD	Coronary heart disease
CVD	Cardiovascular diseases
C2, C8, C14:1	Acylcarnitines
Cer(d18:1/16:0)	Sphingolipids/Ceramides
ET-1	Endothelin-1
ICAM-1	Intercellular adhesion molecule-1
FPA	Fibrinopeptide A
GDF-15	Growth and differentiation factor
GC-MS	Gas Chromatography–Mass Spectrometry
HDMB	Human Database of Metabolomics Biomarkers
HDL	High-density lipoprotein
HPLC	High-performance liquid chromatography
hs-cTnT	High-sensitive troponin T
hs-cTnI	High-sensitive troponin I
LDH	Lactate dehydrogenase
LDL	Low-density lipoprotein
MS	Mass spectrometry
MACE	Major Adverse Cardiac Events
LC-MS/MS	Liquid Chromatography–Tandem Mass Spectrometry
LPC, PE, SM	Lipids/Phospholipids
NO	Nitric oxide
NT-proBNP	Natriuretic peptide
NMR	Nuclear magnetic resonance
PICP	Type I procollagen propeptide
t-PA	Tissue plasminogen activator
RBP4	Retinol-binding protein 4
ST2	Growth stimulation factor
TIMP-1	Tissue inhibitors of matrix metalloproteinases 1
TIMP-2	Tissue inhibitors of matrix metalloproteinases 2
TNF-α	Tumor necrosis factor alpha
sTM	Thrombomodulin
VCAM-1	Vascular cell adhesion molecule-1
VWF	Von Willebrand factor
WHO	World Health Organization
12-HETE, 15-HETE	Oxylipins
